# Managing idiopathic intracranial hypertension in the eye clinic

**DOI:** 10.1038/s41433-024-03140-y

**Published:** 2024-05-24

**Authors:** Laura Bonelli, Vaishnavi Menon, Anthony C. Arnold, Susan P. Mollan

**Affiliations:** 1 Department of Ophthalmology, University of California, Los Angeles, Stein Eye Institute, Los Angeles, CA, USA; 2 Birmingham Neuro-Ophthalmology, University Hospitals Birmingham NHS Foundation Trust, Birmingham, B15 2GW UK; 3 Translational Brain Science, Institute of Metabolism and Systems Research, University of Birmingham, Edgbaston, B15 2TT UK

**Keywords:** Health services, Health care economics, Drug therapy, Education, Prognosis

## Abstract

Idiopathic intracranial hypertension (IIH) is a neuro-ophthalmological condition characterised by a raised intracranial pressure and papilloedema that causes disabling headaches. The main risk factors of female sex and living with obesity have been known for some time, however the knowledge of the underlying pathophysiology is evolving. Papilloedema can impact the visual function, and the majority of people are offered acetazolamide. Those with sight threatening disease need urgent management, though there is little high quality evidence to recommend any particular surgical intervention. Headache treatment is an unmet clinical need and simple medication overuse advice has the potential to reduce the chronification of migraine-like headaches. IIH is emerging as a systemic metabolic disease distinct from people living with obesity alone. While weight loss is the main stay of disease modifying therapy this is challenging to access and many healthcare professionals that manage the condition have no formal training or accessible pathways for weight management. The aim of this “how to do it” article is to present the latest advances in knowledge of IIH that we pragmatically included in routine clinical care for people living with the condition.

Idiopathic intracranial hypertension (IIH) is a syndrome of elevated intracranial pressure (ICP) with specific diagnostic criteria [1, 2]. It occurs most commonly in young women and there is evidence of dysregulated androgens [3]. It is principally associated with obesity [4], however there are systemic metabolic findings which are unique to IIH, that are not conferred by living with obesity alone [5–9]. The incidence of IIH is increasing worldwide [10–12], and as many ophthalmologists now diagnose and manage IIH, high quality research and practice guidelines are required to provide excellence and equity of care [13–15]. Both physicians and patients are central to improving care and identifying those areas which require a change in practice [13, 14, 16]. In this “how to do it” article the foundations of managing IIH in ophthalmology outpatients have been considered through a case based approach. As IIH in childhood (pre-puberty) [17] and IIH without papilloedema [18] are expectantly different conditions, this article will focus on adults with IIH. This is an outline of how IIH is managed in our ophthalmology clinics, though others may prefer a different approach.

## Approaching the history

Patients typically report new onset headaches or a change in their existing headache frequency (approximately 90%) [19, 20]. Early in the disease headaches can be typical of a raised ICP headache that is worse on waking [20–22], however over time, for the majority, they become chronic [23]. These headaches are typically migraine-like and are often accompanied with symptoms of photophobia, phonophobia and nausea [23]. Transient visual obscurations (68%), back pain (53%) and pulsatile tinnitus (52%) are commonly reported [24]. Approximately 33% present with loss of vision [24], and a lower percentage notes diplopia secondary to sixth nerve palsy. Facial nerve palsy is a rare occurrence.

## Making a definite diagnosis

Despite clear diagnostic criteria which are widely accepted [1], there is evidence of diagnostic difficulty for some in confirming a diagnosis of IIH [25]. One of the major stumbling points is the correct identification of papilloedema [25]. Hence a full ophthalmic examination is required to confirm papilloedema and rule out pseudopapilloedema [14, 15]. Common causes of pseudopapilloedema include optic nerve head drusen; anomalous discs; hypermetropia and myopia. Autofluorescence, either from a fundus camera or by optical coherence tomography imaging, can highlight surface drusen. OCT can be helpful, particularly cross sectional imaging to identify buried drusen [26], and peripapillary hyperreflective ovoid mass structures (PHOMS) which may elevate the optic nerve head. Occasionally fundus fluorescein angiography (FFA) might be useful if the diagnosis is uncertain, as early capillary dilation may be observed by followed by late leakage at the optic disc [27, 28].

## Case 1

**Fig. 1:**
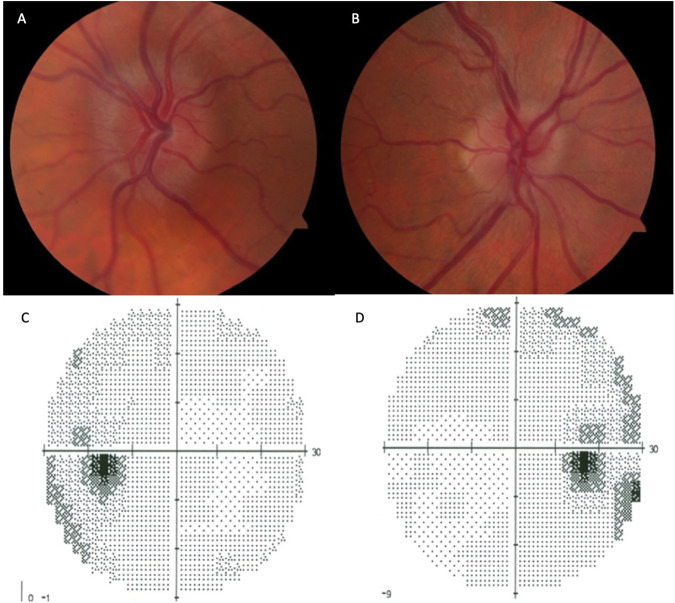
This figure shows Case 1 with mild papilloedema. **A** colour fundus image of the left eye; and **B** right eye. Humphrey 30-2 perimetry showed mild enlargement of the blind spot bilaterally with very mild peripheral changes; **C** grey scale left eye; and **D** right eye.

A 42-year-old woman living with obesity (body mass index (BMI) 46 kg/m^2^), and 23 kg weight gain over the past year, reports persistent daily headaches for the last two months, worse upon awakening in the morning. She denies pulsatile tinnitus, diplopia or changes in vision. She had no recent changes to medications or takes vitamin supplements. On examination, best-corrected visual acuity (BCVA) was 6/6, with all the Ishihara colour plates identified correctly in each eye. Pupils were briskly reactive with no relative afferent pupillary defect (RAPD). Ocular motility was full with no misalignment. Fundoscopy revealed mild bilateral optic nerve oedema (Fig. 1). Humphrey 30-2 perimetry showed mild enlargement of the blind spot bilaterally (Fig. 1).

## Investigations for papilloedema

Following confirmation of true disc oedema, if it has not yet occurred, a full examination of the extent of visual dysfunction should be made. This includes visual acuity, pupil assessment, colour vision and formal visual fields. Bilateral optic nerve oedema with spared visual function is suggestive of papilloedema (optic nerve oedema secondary to elevated ICP). Papilloedema indicates a possible serious underlying condition that requires immediate work up. The first step is to check blood pressure to rule out malignant hypertension. Neuroimaging is then required, and a preferred modality by the authors is magnetic resonance imaging (MRI) of the brain and orbits, with fat suppression technique, with and without contrast. The addition of the orbital imaging allows exclusion of other optic neuropathies, however, has the drawback of a longer examination. MR venogram (MRV) with contrast is performed at the same time. Studies are intended to rule out mass lesion, hydrocephalus, meningeal infiltration, Chiari malformation and venous sinus thrombosis. If MR imaging is not available, the patient has a contraindication or suffers from claustrophobia, a contrast – enhanced computed tomography (CT)/CTV scan is as good an alternative. In this case the brain and orbit MRI with or without contrast showed a partially empty sella and was otherwise unremarkable. Contrast enhanced MRV of the brain was normal, with no evidence of venous sinus thrombosis.

A lumbar puncture (LP) is then a recommended investigation. When discussing with colleagues to arrange the LP, it is important to request that the LP be performed in the left lateral decubitus position and contents taken for protein, glucose and culture. A paired serum glucose should be taken at the same time. The opening pressure should be recorded, and above 25 cm of water (H_2_O) is considered abnormally high [14, 29]. There is controversy over the point at which the LP opening pressure becomes pathological, therefore if the LP opening pressure is between 25 and 30 cm H_2_O most neuro-ophthalmologists may re-evaluate the diagnosis [14, 29]. In case 1 the LP opening pressure was 33 cm H_2_O and cerebrospinal fluid (CSF) constituents were normal. A definite diagnosis of IIH was made as per the diagnostic criteria (Table 1) [1].

**Table 1:** Programmes for weight management in the United Kingdom (correct at time of publication).

Program	Main criteria^a^	Who can refer	Notes
NHS digital weight management programme	18 years old or over; **a BMI greater than 30** **kg/m**^**2**^ (or BMI 27.5 kg/m^2^ for people from Black, Asian, and ethnic minority backgrounds); the person must have diabetes, high blood pressure, or both; and they must have a smartphone, tablet, or computer with internet access	GP or pharmacist	https://www.england.nhs.uk/digital-weight-management/how-to-access-the-programme/ last accessed 24th March 2024
Tier 1 weight management services	Community based and early intervention (self-care)	Self-referral	Tier 1 weight management is a responsibility of public health and local councils. Various community based prevention and early intervention strategies are in place across the region and include self-referrals into franchised slimming companies and referrals to health trainer services
Tier 2 weight management services	**People living with a BMI of over 25** **kg/m**^**2**^ (or over 23 kg/m^2^ if from Black African, African-Caribbean and Asian origin)	Depends on region	Tier 2 services are delivered by local community weight management services, that provide community based diet, nutrition, lifestyle and behaviour change advice, normally in a group setting environment. Normally people can only access these services for a limited time period, often only 12 weeks. For example Birmingham City Council have programmes for people with learning disabilities; people who are visually impaired and people who are physically impaired. Online or face to face e.g. https://www.birmingham.gov.uk/info/50263/supporting_healthier_lives/2480/support_to_manage_weight_tier_2_adult_weight_management_service (last accessed 24th March 2024)
Tier 3 weight management services	**Patients must have a BMI of 40** **kg/m**^**2**^ **or over** (or 35 kg/m^2^ or over if co-morbidities exist). Patients much have engaged with community weight services over a 2 year period (attendance dates should be provided as evidence) and attended a Tier 2 weight management service **unless** BMI is 50 kg/m^2^ or greater^a^	Depends on region, some stipulate GP referral	Clinician led multidisciplinary team (MDT) which may consist of a combination of a specialist nurse, specialist dietitian, psychologist, psychiatrist and physiotherapist. In practice these are the specialist weight management clinics that provide non-surgical, intensive medical management
Tier 4 weight management services^b^	**Patients must have a BMI of 40** **kg/m**^**2**^ **or more, or between 35 and 40** **kg/m**^**2**^ and other significant disease (for example, type 2 diabetes or high blood pressure) that could be improved if they lost weight. All appropriate non-surgical measures have been tried but the person has not achieved or maintained adequate, clinically beneficial weight loss. The person has been receiving or will receive intensive management in a tier 3 service. The person is generally fit for anaesthesia and surgery The person commits to long-term follow-up. **People with a BMI of 50** **kg/m**^**2**^ **or more** Offer an assessment for bariatric surgery to people with a BMI of 50 kg/m^2^ or more as long as they are also receiving or will receive assessment in a tier 3 service.	Tier 3 services or a direct referral (may depend on which region whether they only accept a GP referral)	Severe and complex obesity services (including obesity surgery, obesity medicine MDTs, specialist weight management programmes, post-surgical and annual follow up). It is important to note that tier 4 includes not only bariatric surgery but also bariatric medicine

^a^Note exclusions may apply and some regional criteria may have different BMI categories.

^b^Tier 4 services have other inclusion criteria based for people with live with type 2 diabetes mellitus.

In this investigational phase, it is important to exclude anaemia, as 10% of people with papilloedema will have treatable iron deficiency anaemia [30–32]. Of note if the haemoglobin is pathologically low, not only should investigations for the source of blood loss be investigated, a transfusion of iron and/or blood may be required. Case series have shown that reversal of papilloedema occurs with normalisation of the haemoglobin [30, 31]. A careful drug history should be considered in all, as a systematic review found the strongest relationship with Vitamin A derivatives, tetracycline-class antibiotics, recombinant growth hormone, and lithium, being associated with secondary raised ICP [33]. This almost exclusively occurs in a temporal relationship to commencing the medication.

While the investigations are underway the patient often finds themselves under physicians who may not routinely care for people with IIH or be well versed in ophthalmology. It is therefore critically important that the ophthalmologist is in charge of the vision and re-examines the visual function as, for some, (around 7%), a rapid decline in visual function can occur [11, 34]. Depending on the grade of papilloedema and potential for visual loss, an escalating dose of acetazolamide may be required [14]. Pathways where the patient is discharged from investigations without an assessment on whether medical management is immediately required should not occur.

## Delivering a diagnosis of IIH

Ideally delivering a diagnosis of IIH should be done by professionals with experience of the condition, however it usually happens in non-ideal conditions [16]. It is helpful to gain knowledge of what the patient already knows about their investigations and whether they have heard of IIH. IIH UK, a patient charity, is a useful resource with the ability for the patient to connect with non-medical people for advice. IIH UK have useful patient and healthcare professional information leaflets (these can be found at https://www.iih.org.uk/product/11/2/leaflets (last accessed 28 March 2024)).

## Management of IIH

The main principles of treatment of IIH are to:

1. Protect vision
2. Treat the underlying cause
3. Reduce headache morbidity [14]

## When to use acetazolamide

The use of acetazolamide, a carbonic anhydrase inhibitor, is the most widespread treatment currently used to treat IIH to help with reduction of ICP and to protect the vision. Most physicians start treatment with a dose of between 250 and 500 mg twice a day, and escalate to between 2 g and 4 g in a daily divided dose. In 2014 the Idiopathic Intracranial Hypertension Treatment Trial (IIHTT) [35] provided evidence that acetazolamide, in association with weight loss, was effective in reducing ICP and improving papilloedema in patients with mild to moderate visual field changes and was safe and well tolerated at doses up to 4 g/day. It did not show consistent beneficial effect on headache severity. In cases with improvement of papilloedema and stabilisation of visual fields, continuing headaches may require additional medication. We monitor response to treatment closely initially and titrate as needed. It is fundamental to discuss with the patient the medication’s possible side effects: paraesthesia, dysgeusia (metallic taste), vomiting and diarrhoea, nausea, fatigue, depression and kidney stones. Their knowledge of side effects and strategies to help with its management will help with treatment compliance. Drug labelling recommends monitoring electrolytes periodically while patients are on treatment with acetazolamide, but there is no guidance regarding how frequently. Most patients will develop a chronic compensated metabolic acidosis and potassium decreases mildly.

## When and how to talk about weight management

Central to the care of a person with IIH is a sensitive discussion about the role of body weight and its association with IIH [16]. This should be done by professionals who manage IIH, or preferably by those that manage obesity. A casual conversation of body weight in the emergency setting may do more harm than good [16]. Weight loss methods have evolved over time and the evidence from studies demonstrated good correlation between weight loss and disease remission, however sustained weight loss has not generally been achievable through dietary interventions alone [36].

The IIH weight trial (IIHWT) was a United Kingdom (UK) multicenter randomised controlled trial that evaluated the effect of bariatric surgery and a community weight management intervention on intracranial pressure in women with active IIH and a body mass index of 35 kg/m^2^ or greater [37]. It found that bariatric surgery provided sustained ICP reduction and weight loss for up to two years of follow-up. A health economic analysis was undertaken and this showed by five years, bariatric surgery was more cost-effective than a dietary weight management programme [38]. A per protocol analysis of this trial helped determine how much weight should be lost to ensure disease remission, as defined by normalisation of ICP measured by a lumbar puncture. This was found to be 24% of body weight [39]. This magnitude of weight loss is likely only to be achieved with bariatric surgery [36]. The IIHWT was successful in its primary outcome, however there was no statistical improvement in visual fields. This was likely as it was a cohort of patients with a long duration of the disease [40]. It should also be noted that 24% weight reduction may not be required for remission of papilloedema, where others have found weight reduction of between 5–15% beneficial [41]. A systematic review of weight loss in IIH may be a useful aide [36].

While metabolic and bariatric surgery will deliver additional health benefits, by reversing life-threatening weight related health problems such as certain cancers, cardiovascular disease, and diabetes [42]; there are many barriers to accessing the weight management pathways [43]. In the UK there are various programmes that people with an abnormal BMI potentially may have access to (Table 1): unfortunately, due to supply and demand a number of services may be gapped in certain regions [44].

## How do we treat case 1 with mild optic nerve oedema, normal visual function and headaches?

Start by asking permission to talk about body weight. Explain the condition and association of body weight change and IIH. Then recommending weight reduction (5–15% of current weight as discussed above), and agree how that could be achieved. In addition conservative treatment for headaches may be best, the headaches have only been present for 8 weeks and they may settle once the ICP settles. Advice regarding the use of simple analgesics to treat headaches should be given and highlight that taking too many pain killers could lead to medication overuse headache [22].

In Case 1 with mild optic nerve oedema and normal visual function the joint decision was made to start a weight management programme with a nutritionist, increase physical activity and monitor the clinical signs and symptoms. Should the person have an increase of weight, or escalating symptoms, they should be instructed to contact the clinic for advice.

## How do we monitor patients with IIH?

As ophthalmologists, we have a crucial role in managing patients with IIH. Detailed evaluation to be sure that visual function is preserved and that papilloedema resolves is paramount. Evaluation at regular intervals should include best corrected visual acuity (BCVA), colour vision, pupillary responses and dilated fundus exam. Optic disc photos are useful for documentation and comparison of fundus appearance over time. Formal perimetry is essential to assess optic nerve function. The use of OCT with measurement of peripapillary retinal nerve fibre layer (pRNFL) thickness; disc volume scanning and a macular ganglion cell protocol [45]. The frequency of follow up is determined by the level of optic nerve oedema, optic nerve function, and response to treatment [14].

## Case 1 - outcome

We recommended follow up at 4 weeks, following the first visit. At that time, despite losing 2 kg, visual field and optic nerve appearance had progressed and the patient continued to have headaches. In the IIHWT analysis it was shown that if little or no weight was lost, it could be likely that the disease would progress [39]. She was therefore started on treatment with acetazolamide 500 mg twice a day and we planned for follow up in 4 weeks. At follow up her headaches had improved; and the visual field and optic nerve appearance were back to baseline to mildly improved. Given the good response to treatment, we planned for follow up in 3 months.

## Case 2

**Fig. 2:**
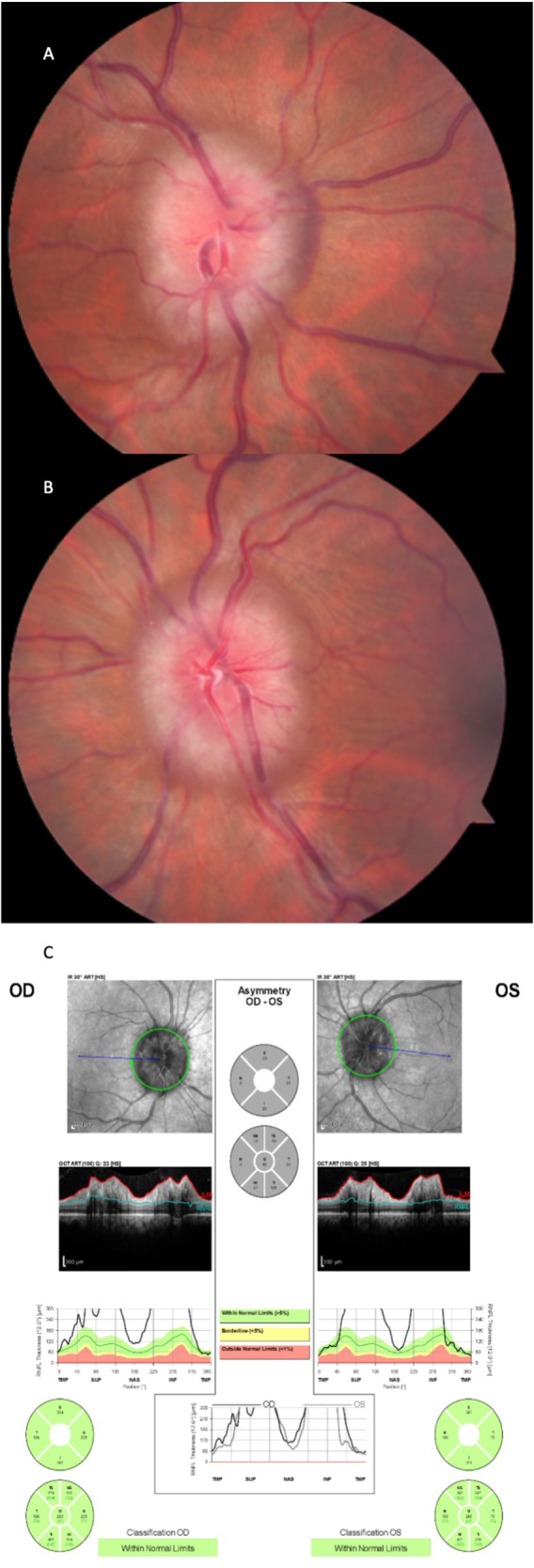
This figure shows Case 2 with moderate papilledema that then has worsening of their visual function and in whom acetazolamide is not tolerated. **A** colour fundus image of the right eye; and **B** left eye. **C** the pRNFL print out with a global RNFL of right eye (OD) 259microns and left eye (OS) 249microns.

**Fig. 3:**
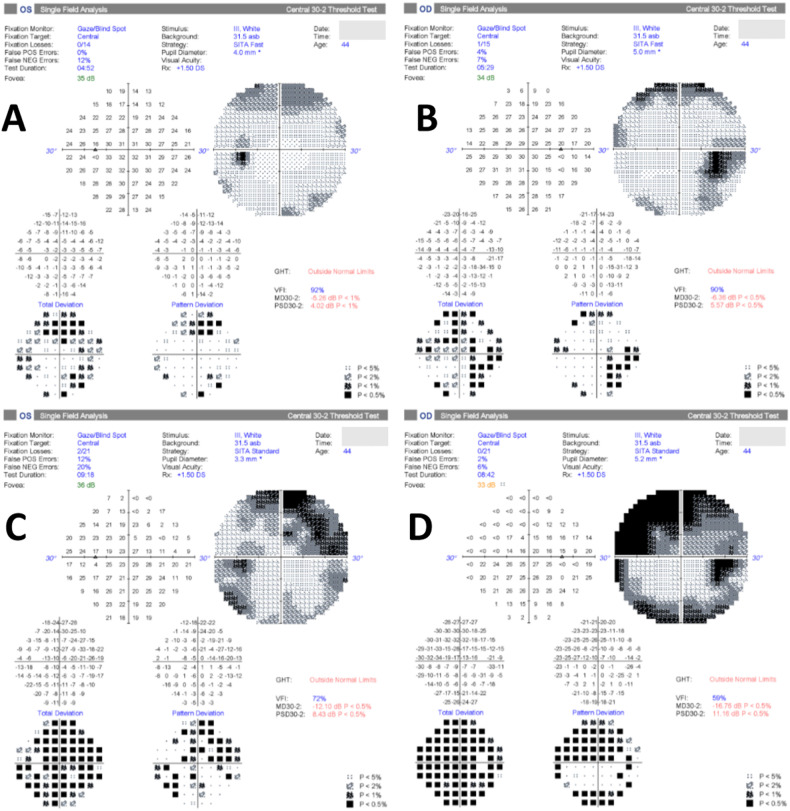
This composite figure shows Case 2 with moderate papilledema that then has worsening of their visual function and in whom acetazolamide is not tolerated. **A** HVF 30-2 of the left eye at the first visit; and **B** HVF 30-2 of the right eye at first visit. Both have enlargement of the blind spots, worse on the right. **C** HVF 30-2 of the left eye at the sight threatening visit; and **D** HVF 30-2 of the right eye when fulminant sight threatening disease is evident with progression of the superior loss and peripheral field constriction.

A 44-year-old African American female with a past medical history of polycystic ovary syndrome (PCOS) and living with obesity (BMI 39 kg/m^2^) was referred for evaluation for papilloedema. She had prior evaluations for headaches diagnosed as migraines; however, treatment was not helping, her vision was becoming blurry and recently she had developed pulsatile tinnitus. Past ocular history was negative. Her medications included ibuprofen 600 mg and sumatriptan 50 mg for migraines, losartan, and atorvastatin. On examination her BCVA was 6/6 in both eyes, colour vision was intact and both pupils were reactive with no RAPD. Dilated fundus exam revealed bilateral optic nerve oedema with obliteration of the physiologic cup and partial obscuration of the disc vessels (Fig. 2). OCT peripapillary RNFL average thickness measured 249microns OD and 219microns OS (Fig. 2). Humphrey 30-2 perimetry showed generalised depression and blind spot enlargement worse in the right eye (Fig. 3). The brain MRI with and without contrast was unremarkable and brain MRV with contrast demonstrated mild bilateral narrowing of the transverse sinus, no evidence of thrombosis was seen. The patient did not have anaemia. The lumbar puncture opening pressure was 45 cm H_2_O and CSF had normal constituents.

## How do we treat case 2 with moderate optic nerve oedema, abnormal visual function and headaches?

Case 2 had moderate compromise of visual function and more severe optic nerve oedema. Treatment included acetazolamide 500 mg twice a day with planned titration up to 1000 mg twice a day over a week and referral to the local weight management programme. Follow up examination two weeks later showed BCVA at 6/6 both eyes, mildly decreased colour vision in the right eye with right RAPD and worsening visual field more significant in the right eye (Fig. 3). The optic nerve oedema had not changed, as evidenced by her OCT imaging. She reported of persistent pulsatile tinnitus and headaches. She had found it difficult to comply with the acetazolamide due to side effects. Due to worsening visual function, persistent severe optic nerve oedema, intolerance to acetazolamide and weight gain in the preceding months, options of intensive weight management or a surgical option to save the sight was discussed with the patient. Together we decided to proceed with surgical management of IIH. The patient underwent placement of a right frontal ventriculoperitoneal shunt (VPS) without complications.

## Case 2 - outcome

Following VPS placement, ocular examination showed BCVA 6/6 in both eyes, right RAPD, improved visual fields and optic nerve oedema. She continued treatment with a weight management programme and eventually underwent bariatric surgery. PCOS is a recognised disease associated with IIH, living with both conditions makes it challenging to sustainably lose weight [46]. The visual and headache outcomes of those with IIH with or without PCOS are similar [47]. Those with PCOS may benefit from targeted weight management with endocrinology. Following bariatric surgery her BMI dropped to 20.5 kg/m^2^ and she did not have a relapse. Visual function and visual fields have remained stable and she has now been discharged.

## Surgical management of IIH

The decision to proceed to surgical management in medically refractory cases should be made on a case by case basis. In this case the visual function was deteriorating rapidly leaving little room for a trial of furosemide or spironolactone, or a very low calorie diet. The decision to proceed to surgery needs to be made jointly with the patient. The surgical management of IIH includes optic nerve sheath fenestration (ONSF), CSF diversion procedures and venous stenting. In most cases the decision is based on the individual patient, each institution’s experience and surgical availability. ONSF is preferred in patients with no significant headache but with severe compromise of vision and persistent optic nerve oedema. It requires an experienced, trained surgeon.

ONSF appears to have less morbidity than CSF diversion procedures. The overall risk of complications is around 10–15%, including a 1–2% risk of loss of vision due to central retinal artery or central retinal vein occlusion (CRAO or CRVO). The most common complications are diplopia, anisocoria and tonic pupil. ONSF does not lower ICP and is not recommended for the treatment of headaches. In some cases, bilateral ONSF is needed to treat papilloedema in the fellow eye [48, 49].

CSF diversion procedures, ventriculoperitoneal shunt and lumboperitoneal shunt, reduce ICP efficiently, leading to resolution of optic nerve oedema and improvement of headaches (in the short term). These procedures require skilled neurosurgeons and can have a number of complications: shunt malfunction, infection, dislocation, which may require re-intervention [49]. In the UK we encourage the use of an ICP monitor, programmable valve and anti-syphon valve for improved outcomes [34, 50–52].

The newest option for treatment is neurovascular stenting [53, 54]. The role of venous stenting in IIH is not clearly defined yet. It may be useful in selected patients with elevated ICP, with venous stenosis and proven elevated pressure gradient, who have failed or cannot tolerate medical treatment. One of the disadvantages is the need for antiplatelet therapy for 6 months following the treatment [54]. There is a UK randomised control trial evaluating the use of stenting in comparison to shunting [55].

## Case 3

**Fig. 4:**
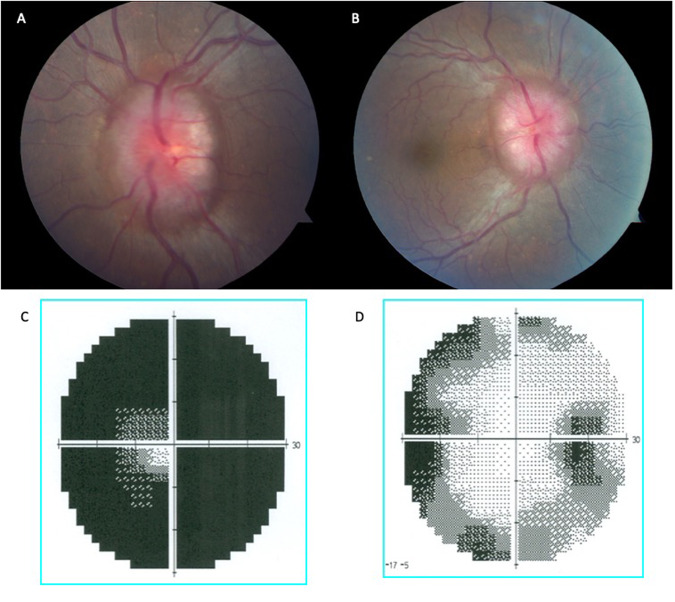
This is a composite figure of Case 3 with severe papilloedema. **A** colour fundus image of the left eye; and **B** right eye. Humphrey 30-2 perimetry showed mild enlargement of the blind spot bilaterally with very mild peripheral changes; **C** grey scale left eye; and **D** right eye.

A 28-year-old female with a history of essential hypertension and living with obesity (BMI 38 kg/m ^2^) presented complaining of darkening vision, worse in the left eye. She reported 3 weeks of progressively worsening severe headache and diplopia. Over the last week she developed frequent transient visual obscurations in both eyes and pulsatile tinnitus. On examination BCVA was 6/9 in the right eye and 6/60 in the left eye. She was able to identify 10/14 Ishihara plates with the right eye and 3/14 with the left eye. Both pupils were sluggish with left RAPD. A right cranial nerve VI palsy was present. Dilated fundus exam revealed severe optic nerve oedema bilaterally (Fig. 4). The global pRNFL thickness was 338microns in the right eye and 334microns in the left eye. Humphrey perimetry 24-2 showed severe generalised depression in both eyes, worse on the left (Fig. 4).

Due to rapidly evolving symptoms of increased ICP and worsening vision over days, along with severe optic nerve oedema, we made the presumptive diagnosis of fulminant IIH. Fulminant IIH is a form of IIH that affects a small percentage of patients [56]. These patients have a very aggressive course with acute onset of symptoms and signs of intracranial hypertension with severe visual loss occurring within weeks of the initial diagnosis or at a relapse of symptoms [57]. Acute presentation with rapid loss of vision is not common and requires ruling out other causes of loss of vision, such as: malignant hypertension, meningitis, cerebral venous disease, optic neuritis (such as myelin oligodendrocyte glycoprotein antibody-associated disease, neuromyelitis optica spectrum disorder, sarcoidosis, syphilis, etc), and optic nerve infiltration (leukaemia, lymphoma). All of which should be picked up in the investigational pathway.

It is important to understand the aggressive nature of this condition and to act immediately. Patients with fulminant IIH should be admitted for expedited work up and treatment. After we ascertained that blood pressure was normal in case 3, we proceeded with work up and treatment. MRI brain and orbit with and without contrast and MRV with and without contrast revealed bilateral enhancement of the optic nerve head and dilation of the optic nerve sheaths, a partially empty sella and narrowing of the transverse sinuses with no evidence of thrombosis. There was no anaemia and no use of precipitating medications. The lumbar puncture opening pressure was 80 cm H_2_O and CSF had a normal composition. Immediate treatment included placement of a lumbar drain, oral acetazolamide 500 mg three times a day to be titrated up to 3–4 g/day rapidly, and intravenous (IV) methylprednisolone 1 g/day for 3 days. Note treatment with steroids is not recommended in IIH with the exception of fulminant cases where a short course of IV methylprednisolone has been more typically used in North American practice, particularly in paediatric cases [58, 59]. In the United Kingdom IV methylprednisolone is not typically used in adults with fulminant IIH. Further evidence would be required to understand the mechanism by which glucocorticoids may be beneficial here.

We consulted with an oculoplastic specialist for possible ONSF and with neurosurgery for possible CSF diversion surgery. It can take time to arrange the emergency surgery, due to the rarity of the surgery. After placement of a lumbar drain, ICP decreased to 50 cm H_2_O but BCVA decreased to counting fingers in the left eye, so the patient underwent left ONSF two days after admission. BCVA remained stable in the right eye at 6/6 and improved to 6/20 in the left eye. Colour vision showed persistent compromise bilaterally. The cranial nerve VI palsy and headaches persisted. Due to the ongoing symptoms and signs and excessively raised ICP the patient finally underwent a right VP shunt placement by neurosurgery four days following the initial admission.

## Case 3 - outcome

The final BCVA was 6/6 in the right eye and 6/24 left eye, with decreased colour vision in both eyes. Cranial nerve VI palsy resolved completely and both optic nerves developed atrophy. Humphrey 30-2 perimetry improved but both eyes had severe peripheral contraction.

## Sight threatening IIH

Thambisetty et al. [58]. published a case series of fulminant IIH. In his series all the patients presented with severe loss of visual acuity, had severe papilledema, visual fields were constricted and the first lumbar puncture showed a mean CSF opening pressure of 54.1 cm H_2_O, with a range between 29 and 70 cm H_2_O. All patients were treated surgically due to rapidly worsening vision. Visual function improved in all patients but despite the aggressive treatment, 50% of them remained legally blind and all of them had residual visual field defects and optic nerve atrophy. Hyder et al. [34]. published a modern case series of patients with sight threatening disease. They noted the more swollen the optic nerve as measured with the initial pRNFL, the impact on long-term visual outcomes was worse. The HVF baseline mean deviation showed that those with a mean deviation worse than −7 dB, did not recover their vision as compared to those whose baseline mean deviation was better than −7 dB. Headache outcomes improved post-surgery however regressed to baseline within 12 months despite ICP normalisation. There has been recent discussion that the definition of fulminant IIH should not be defined by a time from diagnosis to sight threatening disease, but should be defined as the sight threatening disease. This is to ensure that patients do not inadvertently come to harm by a time based definition [57].

## Conclusion

IIH is a complex condition with different degrees of compromise of visual function and headaches that can be debilitating [60]. In this opinion piece we have not fully discussed weight management [16], headache therapies [20, 22], the mental health burden [61] or the maternal health concerns [62]: all of which need to be addressed in a person living with IIH [14, 15]. We have focused on an article for ophthalmologists, where the eye examination determines management. The more severely compromised the visual function, the faster and more aggressively we need to act. Overall, the investigation and management of IIH is a team effort including ophthalmologists, neurologists, neurosurgeons and weight management experts. More patients with IIH will be attending clinic, as the increase in prevalence and incidence is evident [4, 12, 63]. Fortunately, there is new research in this area, with the potential for targeted therapies [64, 65].

## Summary

### What was known before

- Ophthalmologists have a key role in protecting the vision in people who present with papilloedema.
- Incidence and prevalence of Idiopathic Intracranial Hypertension (IIH) is rising.
- The majority of people living with IIH are also living with obesity; there are many barriers to managing weight loss.

### What this review adds

- While IIH is emerging as a metabolic systemic disease that is distinct from obesity, treating obesity can reverse IIH.
- Ophthalmologists can provide basic advice to help reduce headache burden.
- There is a lack of high quality evidence to direct which surgical interventions should be recommended in people with sight threatening disease.

## Data Availability

There is no data regarding this publication.

## References

[1] Friedman DI, Liu GT, Digre KB. Revised diagnostic criteria for the pseudotumor cerebri syndrome in adults and children. Neurology. 2013;81:1159–65. 23966248 10.1212/WNL.0b013e3182a55f17

[2] Yiangou A, Mollan SP, Sinclair AJ. Idiopathic intracranial hypertension: a step change in understanding the disease mechanisms. Nat Rev Neurol. 2023;19:769–85. 37957260 10.1038/s41582-023-00893-0

[3] O’Reilly MW, Westgate CS, Hornby C, Botfield H, Taylor A, Markey K, et al. A unique androgen excess signature in idiopathic intracranial hypertension is linked to cerebrospinal fluid dynamics. JCI Insight. 2019;4:e125348. 30753168 10.1172/jci.insight.125348 PMC6483000

[4] Adderley NJ, Subramanian A, Nirantharakumar K, Yiangou A, Gokhale KM, Mollan SP, et al. Association between idiopathic intracranial hypertension and risk of cardiovascular diseases in women in the United Kingdom. JAMA Neurol. 2019;76:1088–98. 31282950 10.1001/jamaneurol.2019.1812 PMC6618853

[5] Westgate CSJ, Markey K, Mitchell JL, Yiangou A, Singhal R, Stewart P, et al. Increased systemic and adipose 11β-HSD1 activity in idiopathic intracranial hypertension. Eur J Endocrinol. 2022;187:323–33. 35584002 10.1530/EJE-22-0108 PMC9346265

[6] Hardy RS, Botfield H, Markey K, Mitchell J, Alimajstorovic Z, Westgate CSJ, et al. 11βHSD1 Inhibition with AZD4017 improves lipid profiles and lean muscle mass in idiopathic intracranial hypertension. J Clin Endocrinol Metab. 2020;106:174–87. 10.1210/clinem/dgaa766 PMC7765633 33098644

[7] Hornby C, Botfield H, O’Reilly MW, Westgate CSJ, Mitchell J, Mollan SP, et al. Evaluating the fat distribution in idiopathic intracranial hypertension using dual-energy X-ray absorptiometry scanning. Neuroophthalmology. 2018;42:99–104. 29563954 10.1080/01658107.2017.1334218 PMC5858863

[8] Westgate CS, Botfield HF, Alimajstorovic Z, Yiangou A, Walsh M, Smith G, et al. Systemic and adipocyte transcriptional and metabolic dysregulation in idiopathic intracranial hypertension. JCI Insight. 2021;6:e145346. 33848268 10.1172/jci.insight.145346 PMC8262372

[9] Hornby C, Mollan SP, Botfield H, O’Reilly MW, Sinclair AJ. Metabolic concepts in idiopathic intracranial hypertension and their potential for therapeutic intervention. J Neuroophthalmol. 2018;38:522–30. 29985799 10.1097/WNO.0000000000000684 PMC6215484

[10] McCluskey G, Doherty-Allan R, McCarron P, Loftus AM, McCarron LV, Mulholland D, et al. Meta-analysis and systematic review of population-based epidemiological studies in idiopathic intracranial hypertension. Eur J Neurol. 2018;25:1218–27. 29953685 10.1111/ene.13739

[11] Mollan SP, Aguiar M, Evison F, Frew E, Sinclair AJ. The expanding burden of idiopathic intracranial hypertension. Eye. 2019;33:478–85. 30356129 10.1038/s41433-018-0238-5 PMC6460708

[12] Shaia JK, Sharma N, Kumar M, Chu J, Maatouk C, Talcott K, et al. Changes in prevalence of idiopathic intracranial hypertension in the United States between 2015 and 2022, stratified by sex, race, and ethnicity. Neurology. 2024;102:e208036. 38181397 10.1212/WNL.0000000000208036 PMC11097766

[13] Mollan S, Hemmings K, Herd CP, Denton A, Williamson S, Sinclair AJ. What are the research priorities for idiopathic intracranial hypertension? A priority setting partnership between patients and healthcare professionals. BMJ Open. 2019;9:e026573. 30878991 10.1136/bmjopen-2018-026573 PMC6429891

[14] Mollan SP, Davies B, Silver NC, Shaw S, Mallucci CL, Wakerley BR, et al. Idiopathic intracranial hypertension: consensus guidelines on management. J Neurol Neurosurg Psychiatry. 2018;89:1088–1100. 29903905 10.1136/jnnp-2017-317440 PMC6166610

[15] Hoffmann J, Mollan SP, Paemeleire K, Lampl C, Jensen RH, Sinclair AJ. European headache federation guideline on idiopathic intracranial hypertension. J Headache Pain. 2018;19:93. 30298346 10.1186/s10194-018-0919-2 PMC6755569

[16] Abbott S, Denton A, Wong SH, Mollan SP, Bul KC. Weight management communications in idiopathic intracranial hypertension: challenges and recommendations from the patients’ perspective. BMJ Neurol Open. 2023;5:e000527. 38116470 10.1136/bmjno-2023-000527 PMC10729070

[17] Lyons HS, Mollan SLP, Liu GT, Bowman R, Thaller M, Sinclair AJ, et al. Different characteristics of pre-pubertal and post-pubertal idiopathic intracranial hypertension: a narrative review. Neuroophthalmology. 2022;47:63–74. 36891406 10.1080/01658107.2022.2153874 PMC9988343

[18] Mollan SP, Chong YJ, Grech O, Sinclair AJ & Wakerley BR. Current perspectives on idiopathic intracranial hypertension without papilloedema. Life 2021 11, 10.3390/life11060472. 10.3390/life11060472 PMC8225003 34073844

[19] Friedman DI, Quiros PA, Subramanian PS, Mejico LJ, Gao S, McDermott M, Wall M, and the NORDIC IIHTT Study Group. Headache in idiopathic intracranial hypertension: findings from the idiopathic intracranial hypertension treatment trial. Headache. 2017;57:1195–205. 28752894 10.1111/head.13153 PMC5799151

[20] Mollan SP, Grech O, Sinclair AJ. Headache attributed to idiopathic intracranial hypertension and persistent post-idiopathic intracranial hypertension headache: a narrative review. Headache. 2021;61:808–16. 34106464 10.1111/head.14125

[21] Mollan SP, Spitzer D, Nicholl DJ. Raised intracranial pressure in those presenting with headache. BMJ. 2018;363:k3252. 30287521 10.1136/bmj.k3252

[22] Mollan SP, Virdee JS, Bilton EJ, Thaller M, Krishan A, Sinclair AJ. Headache for ophthalmologists: current advances in headache understanding and management. Eye. 2021;35:1574–86. 33580185 10.1038/s41433-021-01421-4 PMC8169696

[23] Mollan SP, Wakerley BR, Alimajstorovic Z, Mitchell J, Ottridge R, Yiangou A, et al. Intracranial pressure directly predicts headache morbidity in idiopathic intracranial hypertension. J Headache Pain. 2021;22:118. 34620087 10.1186/s10194-021-01321-8 PMC8499560

[24] Wall M, Kupersmith MJ, Kieburtz KD, Corbett JJ, Feldon SE, Friedman DI, et al. The idiopathic intracranial hypertension treatment trial: clinical profile at baseline. JAMA Neurol. 2014;71:693–701. 24756302 10.1001/jamaneurol.2014.133 PMC4351808

[25] Fisayo A, Bruce BB, Newman NJ, Biousse V. Overdiagnosis of idiopathic intracranial hypertension. Neurology. 2016 ;86(Jan):341–50. 26718577 10.1212/WNL.0000000000002318 PMC4776085

[26] Shin HJ, Costello F. Imaging the optic nerve with optical coherence tomography. Eye. 2024 *in press*. 10.1038/s41433-024-03165-3 PMC11306400 38961147

[27] Littlewood R, Mollan SP, Pepper IM, Hickman SJ. The utility of fundus fluorescein angiography in neuro-ophthalmology. Neuroophthalmology. 2019;43:217–34. 31528186 10.1080/01658107.2019.1604764 PMC6736131

[28] Pineles SL, Arnold AC. Fluorescein angiographic identification of optic disc drusen with and without optic disc edema. J Neuro-Ophthalmol. 2012;32:17–22. 10.1097/WNO.0b013e31823010b8 PMC3713807 21926917

[29] Mollan SP, Hornby C, Mitchell J, Sinclair AJ. Evaluation and management of adult idiopathic intracranial hypertension. Pr Neurol. 2018;18:485–8. 10.1136/practneurol-2018-002009 PMC6252364 30154235

[30] Mollan SP, Ball AK, Sinclair AJ, Madill SA, Clarke CE, Jacks AS, et al. Idiopathic intracranial hypertension associated with iron deficiency anaemia: a lesson for management. Eur Neurol. 2009;62:105–8. 19521086 10.1159/000222781

[31] Biousse V, Rucker JC, Vignal C, Crassard I, Katz BJ, Newman NJ. Anemia and papilledema. Am J Ophthalmol. 2003;135:437–46. 12654358 10.1016/S0002-9394(02)02062-7

[32] Plant GT, Wong SH, Sundholm A, Mollan SP. Idiopathic intracranial hypertension and anemia: a matched case-control study. J Neuroophthalmol. 2021;41:e272–e273. 33449495 10.1097/WNO.0000000000001183

[33] Tan MG, Worley B, Kim WB, Ten Hove M, Beecker J. Drug-Induced intracranial hypertension: a systematic review and critical assessment of drug-induced causes. Am J Clin Dermatol. 2020;21:163–72. 31741184 10.1007/s40257-019-00485-z

[34] Hyder YF, Homer V, Thaller M, Byrne M, Tsermoulas G, Piccus R, et al. Defining the phenotype and prognosis of people with idiopathic intracranial hypertension after cerebrospinal fluid diversion surgery. Am J Ophthalmol. 2023;250:70–81. 36682516 10.1016/j.ajo.2023.01.016

[35] NORDIC Idiopathic Intracranial Hypertension Study Group Writing Committee, Wall M, McDermott MP, Kieburtz KD, Corbett JJ, Feldon SE, Friedman DI, et al. Effect of acetazolamide on visual function in patients with idiopathic intracranial hypertension and mild visual loss: the idiopathic intracranial hypertension treatment trial. JAMA. 2014;311:1641–51. 24756514 10.1001/jama.2014.3312 PMC4362615

[36] Abbott S, Chan F, Tahrani AA, Wong SH, Campbell FEJ, Parmar C, et al. Weight management interventions for adults with idiopathic intracranial hypertension: a systematic review and practice recommendations. Neurology. 2023;101:e2138–e2150. 37813577 10.1212/WNL.0000000000207866 PMC10663033

[37] Mollan SP, Mitchell JL, Ottridge RS, Aguiar M, Yiangou A, Alimajstorovic Z, et al. Effectiveness of bariatric surgery vs community weight management intervention for the treatment of idiopathic intracranial hypertension: a randomized clinical trial. JAMA Neurol 2021;78:678–86. 33900360 10.1001/jamaneurol.2021.0659 PMC8077040

[38] Elliot L, Frew E, Mollan SP, Mitchell JL, Yiangou A, Alimajstorovic Z, et al. Cost-effectiveness of bariatric surgery versus community weight management to treat obesity-related idiopathic intracranial hypertension: evidence from a single-payer healthcare system. Surg Obes Relat Dis. 2021;17:1310–6. 33952427 10.1016/j.soard.2021.03.020 PMC8241428

[39] Mollan SP, Mitchell JL, Yiangou A, Ottridge RS, Alimajstorovic Z, Cartwright DM, et al. Association of amount of weight lost after bariatric surgery with intracranial pressure in women with idiopathic intracranial hypertension. Neurology. 2022;99:e1090–e1099. 35790425 10.1212/WNL.0000000000200839 PMC9536743

[40] Mollan, Bodoza SP, Ní Mhéalóid S, Mitchell Á, Miller NR JL, Montesano G, et al. Visual field pointwise analysis of the idiopathic intracranial hypertension weight trial (IIH:WT). Transl Vis Sci Technol. 2023;12:1. 37126336 10.1167/tvst.12.5.1 PMC10153590

[41] Daniels AB, Liu GT, Volpe NJ, Galetta SL, Moster ML, Newman NJ, et al. Profiles of obesity, weight gain and quality of life in idiopathic intracranial hypertension (pseudotumor cerebri). Am J Ophthalmol. 2007;143:635–41. 17386271 10.1016/j.ajo.2006.12.040

[42] Gulinac M, Miteva DG, Peshevska-Sekulovska M, Novakov IP, Antovic S, Peruhova M, et al. Long-term effectiveness, outcomes and complications of bariatric surgery. World J Clin Cases. 2023;11:4504–12. 37469732 10.12998/wjcc.v11.i19.4504 PMC10353499

[43] Mollan SP, Tahrani AA, Sinclair AJ. The potentially modifiable risk factor in idiopathic intracranial hypertension: body weight. Neurol Clin Pr. 2021;11:e504–e507. 10.1212/CPJ.0000000000001063 PMC8382420 34484948

[44] NICE Obesity: identification, assessment and management Clinical guideline [CG189] Published: 27 November 2014 Last updated: 26 July 2023.

[45] Thaller M, Homer V, Hyder Y, Yiangou A, Liczkowski A, Fong AW, et al. The idiopathic intracranial hypertension prospective cohort study: evaluation of prognostic factors and outcomes. J Neurol. 2023;270:851–63. 36242625 10.1007/s00415-022-11402-6 PMC9886634

[46] Thaller M, Adderley NJ, Subramanian A, Mollan SP, Sinclair AJ. Co-morbid polycystic ovarian syndrome with idiopathic intracranial hypertension. Neuroophthalmology. 2023;47:49–52. 36798860 10.1080/01658107.2022.2162089 PMC9928479

[47] Thaller M, Homer V, Sassani M, Mollan SP, Sinclair AJ. Longitudinal prospective cohort study evaluating prognosis in idiopathic intracranial hypertension patients with and without comorbid polycystic ovarian syndrome. Eye. 2023;37:3621–8. 37225826 10.1038/s41433-023-02569-x PMC10686374

[48] Subramanian PS, Miller NR. Optic nerve sheath fenestration: does it still have a role in treating patients with elvated intracranial pressure? Clin Exp Ophthalmol. 2023;51:287–8. 37314300 10.1111/ceo.14228

[49] Fonseca PL, Rigamonti D, Miller NR, Subramanian PS. Visual outcomes of surgical intervention for pseudotumor cerebri: optic nerve sheath fenestration versus cerebrospinal fluid diversion. Br J Ophthalmol. 2014;98:1360–3. 24820047 10.1136/bjophthalmol-2014-304953

[50] Tsermoulas G, Thant KZ, Byrne ME, Whiting JL, White AM, Sinclair AJ, et al. The birmingham standardized idiopathic intracranial hypertension shunt protocol: technical note. World Neurosurg. 2022;167:147–51. 36089279 10.1016/j.wneu.2022.08.154

[51] Mollan SP, Momin SNA, Khatkar PS, Grech O, Sinclair AJ, Tsermoulas G. A neuro-ophthalmologist’s guide to advances in intracranial pressure measurements. Eye Brain. 2023;15:113–24. 37790122 10.2147/EB.S404642 PMC10543929

[52] Galloway L, Karia K, White AM, Byrne ME, Sinclair AJ, Mollan SP, et al. Cerebrospinal fluid shunting protocol for idiopathic intracranial hypertension for an improved revision rate. J Neurosurg 2021;136:1790–5. 34624853 10.3171/2021.5.JNS21821

[53] Gurney SP, Ramalingam S, Thomas A, Sinclair AJ, Mollan SP. Exploring the current management idiopathic intracranial hypertension, and understanding the role of dural venous sinus stenting. Eye Brain. 2020;12:1–13. 32021528 10.2147/EB.S193027 PMC6969694

[54] Dinkin MJ, Patsalides A. Idiopathic intracranial venous hypertension: toward a better understanding of venous stenosis and the role of stenting in idiopathic intracranial hypertension. JNO. 2023;43:451–63. 10.1097/WNO.0000000000001898 37410913

[55] IIH Intervention: A clinical trial comparing two treatments (shunts and stents) to preserve vision for people with idiopathic intracranial hypertension ISRCTN57142415 10.1186/ISRCTN57142415. Last accessed 24th March 2024.

[56] Shaia JK, Markle J, Das N, Singh RP, Talcott KE, Cohen D. Characterisation and visual outcomes of fulminant idiopathic intracranial hypertension: a narrative review, Neuro Ophthalmol, 10.1080/01658107.2023.2301358. 10.1080/01658107.2023.2301358 PMC11197909 38933750

[57] Menon V, Stormly-Hansen M, Mollan SP. Fulminant Idiopathic Intracranial Hypertension Should Be Redefined Without a Time Limited Definition: In reply to: Characterisation and visual outcomes of fulminant idiopathic intracranial hypertension: a narrative review. Neuro-Ophthalmology 2024: 1–3. 10.1080/01658107.2024.2337165 10.1080/01658107.2024.2337165 PMC11581150 39583026

[58] Thambisetty M, Lavin PJ, Newman NJ, Biousse V. Fulminant idiopathic intracranial hypertension. Neurology. 2007;68:229–32. 17224579 10.1212/01.wnl.0000251312.19452.ec

[59] Liu GT, Glaser JS, Schatz NJ. High-dose methylprednisolone and acetazolamide for visual loss in pseudotomor cerebri. Am J Ophthalmol. 1994;118:88–96. 8023881 10.1016/S0002-9394(14)72847-8

[60] Adderley NJ, Subramanian A, Perrins M, Nirantharakumar K, Mollan SP, Sinclair AJ. Headache, opiate use, and prescribing trends in women with idiopathic intracranial hypertension: a population-based matched cohort study. Neurology 2022;99:e1968–e1978. 35985824 10.1212/WNL.0000000000201064 PMC9651462

[61] Mollan SP, Subramanian A, Perrins M, Nirantharakumar K, Adderley NJ, Sinclair AJ. Depression and anxiety in women with idiopathic intracranial hypertension compared to migraine: A matched controlled cohort study. Headache 2023;63:290–8. 36748660 10.1111/head.14465 PMC10952318

[62] Thaller M, Homer V, Mollan SP, Sinclair AJ. Disease course and long-term outcomes in pregnant women with idiopathic intracranial hypertension: the iih prospective maternal health study. Neurology 2023;100:e1598–e1610. 36750388 10.1212/WNL.0000000000206854 PMC10103118

[63] Thaller M, Mytton J, Wakerley BR, Mollan SP, Sinclair AJ. Idiopathic intracranial hypertension: evaluation of births and fertility through the Hospital Episode Statistics dataset. BJOG 2022;129:2019–27. 35620863 10.1111/1471-0528.17241 PMC9796176

[64] Mitchell JL, Lyons HS, Walker JK, Yiangou A, Grech O, Alimajstorovic Z, et al. The effect of GLP-1RA exenatide on idiopathic intracranial hypertension: a randomized clinical trial. Brain 2023;146:1821–30. 36907221 10.1093/brain/awad003 PMC10151178

[65] Khatkar P, Hubbard JC, Hill L, Sinclair AJ, Mollan SP. Experimental drugs for the treatment of idiopathic intracranial hypertension (IIH): shedding light on phase I and II trials. Expert Opin Investig Drugs 2023;32:1123–31. 10.1080/13543784.2023.2288073. 38006580 10.1080/13543784.2023.2288073

